# Clustering of health behaviors and their associations with cardiometabolic risk factors among adults at high risk for type 2 diabetes in India: A latent class analysis

**DOI:** 10.1111/1753-0407.13550

**Published:** 2024-05-06

**Authors:** Gabrielli T. de Mello, Sathish Thirunavukkarasu, Panniyammakal Jeemon, Kavumpurathu R. Thankappan, Brian Oldenburg, Yingting Cao

**Affiliations:** ^1^ Research Center for Physical Activity and Health Federal University of Santa Catarina Florianópolis Santa Catarina Brazil; ^2^ Department of Family and Preventive Medicine, School of Medicine Emory University Atlanta Georgia USA; ^3^ Emory Global Diabetes Research Center, Woodruff Health Sciences Center Emory University Atlanta Georgia USA; ^4^ Achutha Menon Centre for Health Science Studies, Sree Chitra Tirunal Institute for Medical Sciences and Technology Trivandrum India; ^5^ Department of Public Health, Amrita Institute of Medical Sciences Kochi Kerala India; ^6^ Baker Heart and Diabetes Institute Melbourne Victoria Australia; ^7^ School of Psychology and Public Health La Trobe University Melbourne Victoria Australia; ^8^ Department of Sport, Exercise and Nutrition Sciences, School of Allied Health, Human Services and Sport La Trobe University Melbourne Victoria Australia

**Keywords:** cardiometabolic risk, clustering, diabetes, health behaviors, latent class

## Abstract

**Background:**

We aimed to identify clusters of health behaviors and study their associations with cardiometabolic risk factors in adults at high risk for type 2 diabetes in India.

**Methods:**

Baseline data from the Kerala Diabetes Prevention Program (*n* = 1000; age 30–60 years) were used for this study. Information on physical activity (PA), sedentary behavior, fruit and vegetable intake, sleep, and alcohol and tobacco use was collected using questionnaires. Blood pressure, waist circumference, 2‐h plasma glucose, high‐density lipoprotein and low‐density lipoprotein cholesterol, and triglycerides were measured using standardized protocols. Latent class analysis was used to identify clusters of health behaviors, and multilevel mixed‐effects linear regression was employed to examine their associations with cardiometabolic risk factors.

**Results:**

Two classes were identified, with 87.4% of participants in class 1 and 12.6% in class 2. Participants in both classes had a high probability of not engaging in leisure‐time PA (0.80 for class 1; 0.73 for class 2) and consuming <5 servings of fruit and vegetables per day (0.70 for class 1; 0.63 for class 2). However, participants in class 1 had a lower probability of sitting for >=3 h per day (0.26 vs 0.42), tobacco use (0.10 vs 0.75), and alcohol use (0.08 vs 1.00) compared to those in class 2. Class 1 had a significantly lower mean systolic blood pressure (*β* = −3.70 mm Hg, 95% confidence interval [CI] −7.05, −0.36), diastolic blood pressure (*β* = −2.45 mm Hg, 95% CI −4.74, −0.16), and triglycerides (*β* = −0.81 mg/dL, 95% CI −0.75, −0.89).

**Conclusion:**

Implementing intervention strategies, tailored to cluster‐specific health behaviors, is required for the effective prevention of cardiometabolic disorders among high‐risk adults for type 2 diabetes.

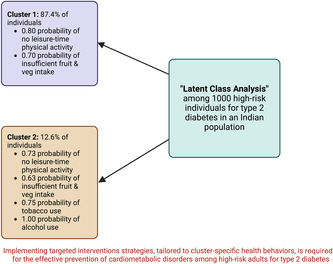

## Graphical abstract

Highlights

- Two clusters of health behaviors were identified in a high‐risk population for type 2 diabetes in an Indian setting.
- Individuals in cluster 1 (87.4% of participants) exhibited a high probability of not engaging in leisure‐time physical activity and consuming insufficient amounts of fruits and vegetables. This pattern was also observed in cluster 2 (12.6% of participants) in addition to having a high likelihood for tobacco and alcohol use.
- These findings emphasize the importance of interventions targeting cluster‐specific health behaviors to prevent cardiometabolic disorders among high‐risk individuals for type 2 diabetes.

## 1 INTRODUCTION

Cardiometabolic risk factors significantly increase the likelihood of developing cardiovascular disease (CVD) and diabetes.^1^ CVD accounted for 17.8 million deaths globally in 2017; more than 80% of these deaths occurred in low‐ and middle‐income countries (LMICs) such as India.^2^ An estimated half a billion adults in the world have diabetes (>90% type 2 diabetes), and this number is projected to increase to 783 million by 2045.^3^ The burden of CVD and diabetes is driven by key cardiometabolic risk factors, including obesity, hypertension, diabetes, and abnormal lipid levels.^1^ These cardiometabolic risk factors, in turn, are strongly linked to health behaviors, including insufficient physical activity (PA), sedentary behavior, inadequate sleep, unhealthy diet, excessive alcohol consumption, and tobacco use.^3^, ^4^, ^5^, ^6^

Health behaviors do not occur in isolation; studies suggest that they cooccur among adults in patterns or clusters.^7^, ^8^ A systematic review of 56 studies conducted among adults worldwide found that the most frequent clustering was characterized by “alcohol use and smoking,” followed by “poor diet with low PA.”^8^ Another systematic review of 37 studies conducted among the general population of the United Kingdom showed that there was strong evidence of clustering between “alcohol misuse and smoking” and “unhealthy diet and smoking.”^9^ In addition, this systematic review reported that the clustering of these behaviors differs by sex. For example, combined drug use, smoking, and alcohol use were more frequently seen in men than women.^9^ Also, studies have reported that men were more likely to present with clustering characterized by a higher number of risky health behaviors compared with women.^7^, ^9^, ^10^

Studies have demonstrated that the clustering of healthy behaviors is associated with a lower risk of CVD and all‐cause mortality.^11^, ^12^ A multicenter study in Spain showed that adults belonging to “healthy PA” and “healthy diet” behavior clusters were less likely to have a lower waist circumference, body fat percent, systolic blood pressure (SBP), and cardiovascular risk compared to people in clusters characterized by reduced PA levels.^8^ Similar results were also found in a study from the United States showing that youth in clusters with the highest average minutes of PA had lower levels of SBP, glucose, and insulin compared with those in the least physically active cluster.^13^ A study conducted on Australian adults who met PA recommendation but spent longer time watching television (TV) (>2.6 h per day for men; >2.1 h per day for women) had high levels of waist circumference, SBP, 2‐h plasma glucose, and triglycerides.^14^ This suggests that even those who meet daily recommended levels of PA could present with worse cardiometabolic risk when other risk behaviors, such as high TV viewing time, cooccur. Interestingly, another study using the same Australian cohort demonstrated that those with the highest level of prudent diet and low TV‐viewing time had the lowest 2‐h plasma glucose compared to their counterparts.^15^ These research findings highlight the varying impact of different combinations or clusters of health behaviors on cardiometabolic risk factors across different populations.

Understanding the cooccurrence of health behaviors could help develop intervention strategies addressing multiple behaviors to alleviate a range of cardiometabolic risk factors and prevent the development of cardiometabolic disorders.^7^, ^9^ The majority of studies investigating the clustering of health behaviors and their associations with cardiometabolic risk factors in adults have been conducted in high‐income countries (HICs).^7^, ^9^ However, none have comprehensively evaluated these relationships in high‐risk populations for type 2 diabetes in LMICs. There is a need for studies in LMICs because social, economic, cultural, and demographic factors affect individual behaviors differently in different populations,^16^, ^17^ and so findings from HIC studies cannot be generalized to LMIC populations. Therefore, the aims of this study were (a) to identify the clustering of health behaviors, including PA, sedentary behavior, diet, sleep, and alcohol and tobacco use among adults at high risk for diabetes in an Indian population; and (b) to examine the associations between these clusters and cardiometabolic risk factors.

## 2 METHODS

### 2.1 Study design, setting, and population

For this analysis, we used baseline data collected in 2013 from participants in the cluster randomized controlled trial of the Kerala Diabetes Prevention Program (K‐DPP). The K‐DPP study, extensively described elsewhere,^18^, ^19^, ^20^, ^21^, ^22^ was a peer‐support lifestyle‐based diabetes prevention program conducted among adults at high risk for type 2 diabetes in Kerala state, India. The trial was conducted in 60 randomly selected polling areas (electoral divisions) in Neyyattinkara taluk (subdistrict) of Trivandrum district from 2013 to 2016. A total of 3689 participants aged 30–60 years were contacted at their households and screened with eligibility criteria. Those satisfying the criteria (*n* = 2586) were further screened using the Indian Diabetes Risk Score (IDRS).^23^ Individuals with an IDRS score of ≥60 (*n* = 1529) were invited to undergo a 2‐h 75‐g oral glucose tolerance test in community‐based clinics. Among the 1209 individuals who attended the clinics, 202 were diagnosed with type 2 diabetes^24^ and were excluded from the study. The remaining 1007 high‐risk individuals (IDRS ≥60 without type 2 diabetes) were enrolled in the trial.^25^ For this analysis, all required data were available for 1000 participants (99.3%).

### 2.2 Procedures and measures

Information on measurement tools, questionnaires, protocols, and data collection process is detailed elsewhere.^18^ Standard questionnaires were administered, and physical measurements were taken by trained staff.

#### 2.2.1 Behavioral variables

We considered six health behaviors for this analysis, encompassing physical activity, sedentary behavior, fruit and vegetable intake, sleep hours, and tobacco and alcohol use. These specific health behaviors were included based on their established associations with cardiometabolic disorders and risk factors among adults.^26^, ^27^, ^28^, ^29^, ^30^, ^31^ The Global Physical Activity Questionnaire^32^ was used to obtain data on leisure‐time PA and sedentary behavior. For leisure‐time PA, participants answered the question, “Do you do any vigorous‐ or moderate‐intense sports, fitness or recreational (leisure) activities that cause large increases in breathing or heart rate like [running or football] for at least 10 minutes continuously?” (Answer options: yes/no). Sedentary behavior was measured by the question, “How much time do you usually spend sitting or reclining on a typical day?”. Data on fruit and vegetable consumption were collected using a food frequency questionnaire adapted from a previous study conducted in Kerala.^33^ Participants reported hours per night sleeping through the question “How many hours do you usually sleep a night?” Tobacco and alcohol use (yes/no) were measured with the questions from the World Health Organization STEPwise approach to NCD risk factor surveillance (WHO STEPS) questionnaire^34^: (a) Did you use any of the following tobacco products (smoking: cigarettes, bidis, cigars and hookah; smokeless: snuff, betel with tobacco, khaini, and gutka) in the last 30 days?; and (b) Did you consume an alcoholic drink (such as beer, wine, whiskey, toddy) in the last 30 days?

#### 2.2.2 Cardiometabolic variables

Seven key cardiometabolic variables were considered for this analysis, namely systolic blood pressure (SBP), diastolic blood pressure (DBP), waist circumference, 2‐h plasma glucose, high‐density lipoprotein (HDL) and low‐density lipoprotein (LDL) cholesterol, and triglycerides.^35^ Blood pressure was measured after a 5‐min resting period three times on the right arm in a seated position using the Omron automatic blood pressure monitor.^34^ The mean of the second and third measurements were used for analyses. Waist circumference was measured midway between the lowest rib and the iliac crest using an inelastic Seca tape.^34^ Blood samples were centrifuged within 30 min of collection and transported in boxes with dry ice to a nationally accredited laboratory. Plasma glucose was measured using the hexokinase method on a COBAS 6000 analyzer with kits supplied by Roche Diagnostics, Switzerland. Lipids were measured by enzymatic methods on a COBAS 6000 analyzer using kits provided by Roche Diagnostics, Switzerland. LDL cholesterol was estimated using the Friedewald equation^36^ for participants with triglycerides ≤4.52 mmol/L, and for the rest, values obtained from the direct method were used in the analysis.

#### 2.2.3 Sociodemographic variables

Sociodemographic variables included age (years), sex, occupation (skilled/unskilled and homemaker/unemployed/retired), marital status (married and separated/divorced/widowed/never married), and level of education (low up to primary, middle [middle school, secondary, and higher secondary], and High [college and above]).

### 2.3 Statistical analysis

Participants' characteristics were described using means and SDs for continuous variables and relative and absolute frequencies for categorical variables. The clustering of health behaviors was identified with latent class analysis (LCA)^37^ using the poLCA package in R software. LCA models were applied using six health behaviors: (a) leisure‐time PA (yes/no); (b) sitting time (<3 and ≥3h/day)^38^; (c) servings of fruit and vegetables per day (<5 and ≥5)^34^; (d) hours of sleep in the night (<7 and ≥7)^39^; (e) tobacco use (yes/no); and (f) alcohol use (yes/no). Models with two (model 1) to six classes (model 5) were generated. Bayesian information criterion (BIC), Akaike information criterion (AIC), consistent AIC (CAIC), adjusted BIC (ABIC), and log‐likelihood ratios were used to compare the models to select the model with the best fit and more parsimonious than the other models.^37^ Participants were allocated to a class according to the highest probability of belonging to a class as determined by individual behavior patterns.^37^

Multilevel mixed‐effects linear regression^40^ was used to examine the associations between clusters of health behaviors (identified by LCA) and cardiometabolic risk factors, including SBP and DBP, waist circumference, 2‐h plasma glucose, HDL and LDL cholesterol, and triglycerides. Triglycerides were log‐transformed as the data were skewed. Clusters (polling areas) were considered as level two in the models. Models were adjusted for age, sex, marital status, education, and occupation. The results are expressed as β‐coefficients with their respective 95% confidence intervals (95% CIs). Association analyses were conducted in Stata version 17.0 (Stata Inc., College Station, TX, USA).

## 3 RESULTS

Table 1 shows the characteristics of the participants. The mean age was 46.0 (SD 7.5) years, and 52.6% were male. The majority were married (95.1%), educated between middle and higher secondary school (58.9%), and engaged in paid labor (72.1%). With regard to health behaviors, slightly more than three fourths (79.4%) and two thirds (69.0%) reported not engaging in leisure‐time physical activity and consuming <5 servings of fruit and vegetables per day, respectively. The prevalence of tobacco and alcohol use was around 20%, with a higher proportion of males (34.0% and 39.2%, respectively) reporting the use than females (2.3% and 0.4%, respectively). The mean SBP was in the prehypertension range (123.3 mm Hg), the mean waist circumference was in the central obesity range in females (87.0 cm), and the mean LDL cholesterol (154.6 mg/dL) was more than optimal. All other cardiometabolic variables were within the normal range.

**TABLE 1:** Characteristics of study participants.

Variables	Overall (*N* = 1007)
Sociodemographics	
Age (years), mean (SD)	46.0 (7.5)
Sex, *n* (%)	
Female	474 (47.4)
Male	526 (52.6)
Marital status, *n* (%)	
Married	951 (95.1)
Single^a^	49 (4.9)
Education, *n* (%)	
Low (up to primary school)	251 (25.1)
Middle (middle to higher sec. school)	589 (58.9)
High (college and above)	160 (16.0)
Occupation, *n* (%)	
Skilled/unskilled	721 (72.1)
Homemaker	268 (26.8)
Unemployed/retired	11 (1.1)
Health behaviors	
Leisure‐time physical activity, n (%)	
Yes	206 (20.6)
No	794 (79.4)
Sitting time (hours/day), *n* (%)	
< 3	714 (71.4)
≥ 3	286 (28.6)
Fruit and vegetable servings/day, *n* (%)	
< 5	690 (69.0)
≥ 5	310 (31.0)
Hours of sleep in night, *n* (%)	
< 7	445 (44.5)
≥ 7	555 (55.5)
Tobacco use, *n* (%)	
Overall	190 (19.0)
Male	179 (34.0)
Female	11 (2.3)
Alcohol use, *n* (%)	
Overall	209 (20.8)
Male	206 (39.2)
Female	2 (0.4)
Cardiometabolic risk factors	
Systolic BP (mm Hg), mean (SD)	123.3 (17.7)
Diastolic BP (mm Hg), mean (SD)	74.9 (11.8)
Waist circumference (cm), mean (SD)	88.3 (9.7)
Male (*n* = 526)	89.5 (9.3)
Female (*n* = 474)	87.0 (10.0)
2‐h plasma glucose (mg/dL), mean (SD)	107.0 (28.1)
HDL cholesterol (mg/dL), mean (SD)	51.0 (15.1)
Male	54.0 (14.3)
Female	48.4 (15.3)
LDL cholesterol (mg/dL), mean (SD)	154.6 (36.1)
Triglycerides (mg/dL), median (IQR)	100.5 (77.0–139.5)

Abbreviations: BP, blood pressure; HDL, high‐density lipoprotein; IQR, interquartile range; LDL, low‐density lipoprotein.

^a^ Single includes separated, divorced, widowed, and never married.

LCA parameters (Table 2) and item‐response probabilities (Table 3) of models revealed 2–6 latent classes. Model 1, with two classes, was considered the best fit, as it had the lowest BIC, ABIC, and CAIC values (Table 2). Model 1 was also more parsimonious than the other models. Two classes (or clusters) were identified, with most participants belonging to class 1 (*n* = 874, 87.4%). Both classes were characterized by a high probability of not engaging in leisure‐time PA (0.80 for class 1; 0.73 for class 2) and consuming <5 servings of fruit and vegetables per day (0.70 for class; 0.63 for class 2). The probability of sleeping for ≥7 h per night was similar between the two classes (0.56 for class 1; 0.52 for class 2). On the other hand, compared with participants in class 2, those in class 1 had a lower probability of sitting for 3 or more hours per day (0.42 vs 0.26), using tobacco (0.75 vs 0.10), and drinking alcohol (1.00 vs 0.08).

**TABLE 2:** Parameters of the latent class analysis models.

Models	Log‐likelihood	Degrees of freedom	BIC	ABIC	CAIC	Likelihood ratio
Overall (*N* = 1000)						
Model 1	−3315.672	50	6721.17	6679.88	6734.17	63.09
Model 2	−3306.626	43	6751.45	6687.93	6771.45	45.00
Model 3	−3301.263	36	6789.09	6703.34	6816.09	34.27
Model 4	−3296.817	29	6828.57	6720.58	6862.57	25.38
Model 5	−3296.817	22	6871.37	6741.15	6912.37	19.81

Abbreviations: ABIC, adjusted Bayesian information criterion; BIC, Bayesian information criterion; CAIC, consistent Akaike information criterion.

**TABLE 3:** Prevalence and item‐response probabilities of each behavior for each class.

Prevalence and item‐response probabilities	Overall (*N* = 1000)	
	Classes	
	1	2
Prevalence	87.4%	12.6%
Item‐response probabilities		
Leisure‐time physical activity		
No	0.80	0.73
Yes	0.20	0.27
Sitting time (hours/day)		
<3	0.74	0.58
≥3	0.26	0.42
Sleep (hours/night)		
<7	0.44	0.48
≥7	0.56	0.52
Fruits and vegetables (servings/day)		
<5	0.70	0.63
≥5	0.30	0.37
Tobacco use		
Yes	0.10	0.75
No	0.90	0.25
Alcohol use		
Yes	0.08	1.00
No	0.93	0.00

Class 1 had lower mean SBP (*β* = −3.70 mm Hg, 95% CI −7.05, −0.36), DBP (*β* = −2.45 mm Hg, 95% CI −4.74, −0.16), and triglycerides (*β* = −0.81 mg/dL, 95% CI −0.89, −0.75) as compared to class 2, after adjusting for age, sex, education, marital status, and occupation in multilevel mixed‐effects models (Table 4).

**TABLE 4:** Associations between clusters of health behaviors and cardiometabolic risk factors.

Variables	Overall (*N* = 1000)		*p* value
	β	95% CI	
Systolic blood pressure (mm Hg)			
Class 2 (*n* = 874)	Ref.	‐	‐
Class 1 (*n* = 126)	−3.70	−7.05, −0.36	.030
Diastolic blood pressure (mm Hg)			
Class 2 (*n* = 874)	Ref.	‐	‐
Class 1 (*n* = 126)	−2.45	−4.74, −0.16	.036
Waist circumference (cm)			
Class 2 (*n* = 874)	Ref.	‐	‐
Class 1 *(n* = 126)	0.42	−1.46, 2.30	.66
2‐h plasma glucose (mg/dL)			
Class 2 (*n* = 874)	Ref.	‐	‐
Class 1 (*n* = 126)	4.35	−1.23, 9.92	.13
HDL cholesterol (mg/dL)			
Class 2 (*n* = 874)	Ref.	‐	‐
Class 1 (*n* = 126)	−1.99	−4.91, 0.94	.18
LDL cholesterol (mg/dL)			
Class 2 (*n* = 874)	Ref.	‐	‐
Class 1 (*n* = 126)	−2.25	−9.48, 4.98	.54
Triglycerides (mg/dL)			
Class 2 (*n* = 874)	Ref.	‐	‐
Class 1 (*n* = 126)	−0.81	−0.75, −0.89	<.001

*Note*: Analyses were adjusted for age, sex, education, marital status, and occupation in multilevel mixed‐effects linear regression models. Clusters (polling areas) were considered as level two in the models.

Abbreviations: CI, confidence interval; HDL, high‐density lipoprotein; LDL, low‐density lipoprotein.

## 4 DISCUSSION

This study identified two behavior clusters (or classes) using LCA of six common health behaviors in Indian adults at high risk for type 2 diabetes. Nearly 90% of the study participants were allocated to class 1, characterized by a higher probability of no leisure‐time physical activity, consuming <5 servings of fruit and vegetables per day, and a lower likelihood of sedentary behavior and tobacco and alcohol use. Participants in class 1 had a lower mean SBP, DBP, and triglycerides than those in class 2.

Our study findings align with previous research conducted among general adult populations.^7^, ^9^, ^41^ The existing literature indicates that the most prevalent clusters of health behaviors among adults involve low fruit and vegetable intake combined with low physical activity, as well as alcohol drinking and smoking. Specifically, the highest prevalence, ranging from 47% to 54%, was observed for the cluster characterized by “low fruit and vegetable intake and low physical activity.” Furthermore, the prevalence ratios were highest for alcohol use and smoking, ranging from 1.89 to 2.89.^7^, ^9^

Class 1 had lower levels of mean blood pressure and triglycerides when compared to class 2. Similar results were found in the EVIDENT study from Spain conducted among 1327 adults (aged 20–80 years).^8^ In this multicenter study, the “healthy/PA” and “healthy/diet” clusters had better SBP and cholesterol levels than the “unhealthy cluster” (more sedentary, high smoking, and worse clinical parameters).^8^ Clearly, these findings suggest that adopting a high number of healthy behaviors results in a better cardiometabolic risk profile, potentially reducing the risk of developing cardiometabolic diseases.

Identifying two distinct clusters of health behaviors within our study population emphasizes the importance of implementing targeted intervention strategies.^7^, ^9^ Prioritizing initiatives to enhance leisure‐time physical activity and promote increased fruit and vegetable intake is crucial for individuals in both identified classes. Furthermore, specific efforts should be directed toward tobacco cessation and reducing alcohol consumption, especially for those categorized in class 2.

A major strength of this paper is the large sample size recruited from the community. Another strength is that we applied the LCA procedure in an under‐studied LMIC population. Using LCA, the classes identified are not based on assumption but on latent variables (unobserved), promoting less arbitrary decisions in forming clusters.^37^ In addition, the study was conducted in Kerala, the state with the highest prevalence of type 2 diabetes and several other cardiometabolic risk factors in India,^42^, ^43^ as the state is in the most advanced stage of epidemiological transition.^44^ Therefore, Kerala is the precursor for the rest of India regarding the burden of cardiometabolic disorders,^44^ making it an ideal setting for this analysis. Finally, the completeness of variables used in the analyses was almost 100%.

Some limitations also need to be considered. Data on health behaviors were self‐reported. However, we used standard questionnaires that were pilot tested and administered by highly trained research staff. The study findings apply only to those at high risk of developing diabetes but not to the general population whose health behaviors may differ from those of the high‐risk individuals. A;though we acknowledge that our study data are a decade old, recent studies (in 2018 and 2019) conducted among adults in Kerala have consistently demonstrated a high prevalence of low physical activity, insufficient intake of fruits and vegetables, tobacco use, and alcohol use.^45^, ^46^, ^47^, ^48^ These contemporary findings mirror the patterns observed a decade ago among adults in Kerala.^21^, ^42^, ^43^, ^49^ Consequently, we anticipate that similar clusters of health behaviors would emerge in the study population if analyzed using data collected in the current period. Importantly, we have successfully followed up with 85% of the original K‐DPP trial participants in 2022, and there are concrete plans in place to conduct a longitudinal cluster analysis of health behaviors covering 10 years (from 2013 to 2022). Finally, this is a cross‐sectional study, and thus, causality could not be established for the association between clusters of health behaviors and cardiometabolic risk factors.

In conclusion, this study identified two distinct clusters of behavioral patterns within a high‐risk Indian population for type 2 diabetes. Both clusters exhibited a high likelihood of not engaging in leisure‐time physical activity and consuming inadequate amounts of fruits and vegetables. Furthermore, tobacco use and alcohol consumption were notably prevalent among individuals classified under cluster 2. These findings could help design health promotion interventions targeting cluster‐specific health behaviors to prevent cardiometabolic disorders among high‐risk individuals for type 2 diabetes.

## FUNDING INFORMATION

The original K‐DPP study was supported by funding from the National Health and Medical Research Council (1005324) and the Fogarty International Centre (D43TW008332). The funders had no role in study design, data collection and analysis, decision to publish, or preparation of the manuscript. ST was partially supported by grant #75D30120P0742 from the Centers for Disease Control and Prevention (CDC) Atlanta.

## CONFLICT OF INTEREST STATEMENT

No authors have potential conflicts of interest related to this research work.

## Data Availability

Data of this study are available from the corresponding author (Gabrielli Thais de Mello) upon reasonable request and contingent on ethics committee approval.
